# Spinal cord stimulation may reduce lumbar radiculopathy in the setting of metastatic colon cancer

**DOI:** 10.1016/j.inpm.2023.100374

**Published:** 2023-12-15

**Authors:** Harman Chopra, Melissa Jackels, Arvind Senthil Kumar, Mustafa Broachwala, Tariq AlFarra, Joel Castellanos

**Affiliations:** a Physical Medicine & Rehabilitation, Johns Hopkins University School of Medicine, Baltimore, MD, USA; b Division of Pain Medicine, University of California San Diego Medical Center San Diego, CA, USA

## Abstract

Cancer pain has a substantial impact on the quality of life and functional capacity with a prevalence of up to 70 % in patients with advanced, metastatic, or terminal disease [1]. The WHO pain ladder has been used in practice to guide cancer pain management. A three-step ladder starts with NSAIDs and non-opioids for mild pain, weak opioids for mild to moderate pain and strong opioids for moderate to severe pain with the use of adjuvant medications such as TCAs and muscle relaxants at any stage for optimization (Fallon et al., Dec 2022) [2]

We present a case of a patient with metastatic colon cancer who was admitted for intractable pain crisis and right sided L-5 radiculopathy secondary to epidural metastasis (Figs. 1 and 2). The patient's pain left her bedridden, unable to walk and remained refractory to an escalating intravenous opioid regimen and caudal epidural steroids. The patient subsequently underwent spinal cord stimulation (SCS) trial at level T-7 and achieved >80 % pain relief resulting in a markedly decreased opioid requirement and tremendous recovery of ambulatory function (Fig. 3). After sustained results, a permanent implant was placed at T-8 and patient remains discharged with functional restoration and continued pain improvement (Fig. 4).

To our knowledge, this is a novel application of SCS for a refractory pain crisis secondary to a metastatic colon cancer induced radiculopathy presenting with severe functional impairment. As we transition away from opioid use, it is imperative as pain physicians, to investigate the potential of current as an alternative means of cancer pain management: a ubiquitous and challenging clinical conundrum.

## 1 Introduction

Over 70 % of cancer patients with advanced disease suffer from malignancy-related pain, and over half of them experience undertreatment, leading to significant morbidity and functional decline [1]. Recent literature has shown that adequate pain control correlates with better outcomes and quality of life (QoL) [2,3]. Cancer pain can be categorized into somatic, visceral and neuropathic. Neuropathic cancer pain (NCP) is typically the result of direct nerve infiltration or mechanical compression by the cancer. Once a peripheral nerve is damaged, its fibers become hypersensitive which alters conduction and makes it prone to spontaneous discharges, characterized in patients as allodynia, an exaggerated pain response to a mild stimulus [4]. NCP is characterized by the classic “burning or electrical” sensation, however, it can sometimes manifest with myelopathy or radiculopathy presenting with decreased sensation or muscle weakness [5]. It is a chronic, fluctuating issue that persists in the daily lives of cancer patients, with acute breakthrough episodes of severe pain, sometimes several times a day. Episodes of breakthrough pain can be spontaneous, and triggers can be unclear but can include position, temperature or even touch.

Opioids remain the mainstay of treatment for severe cancer pain, but up to 20 % of patients have persistent or refractory pain despite rapid and aggressive opioid titration or develop refractory pain after chronic opioid use. Another obstacle is that opioids remain suboptimal in the treatment of neuropathic pain. NCP has a complex pathophysiology, and its mechanisms involve different regions of the central nervous system and specified structures of the peripheral nervous system. An injury in any one of these regions can trigger a cascade, culminating in the phenomena of peripheral and central sensitization [6]. This is where alternative procedures such as SCS should be explored.

SCS, first developed in 1967 by Shealy, came to fruition after the discovery of the “gate-control theory” which provided a mechanism for pain transmission at the junction between peripheral and central nerves [7,8]. This is a therapeutic neuromodulation in which percutaneously implanted leads deliver an electric current at a controlled frequency and intensity, which selectively disrupts the pathologic, hyperactive pain circuits traveling to the brain via the spinothalamic tracts [9]. Typically, patients who fail conservative standard of care undergo placement of temporary percutaneous leads or “trial leads”. A successful SCS trial is defined as a reduction of pain by 50 % and functional improvement in patient's ability to complete activities of daily living (ADLs, and is required prior to consideration for permanent implant [10].

Tonic stimulation is a type of SCS which utilizes consistent, low frequency stimulation to induce paresthesias which modulate the perception of pain in the affected regions. Tonic stimulation has historically been the conventional waveform used to treat neuropathic pain syndromes and offers a potential solution to address malignancy-related pain [6]. The literature documenting SCS as a treatment modality for cancer related neuropathic pain is represented with case reports and small case series. In these reports, authors describe patients reporting substantial pain relief, decrease in daily requirements of pain medications and improved functional parameters.

## 2 Case report

**Fig. 1:**
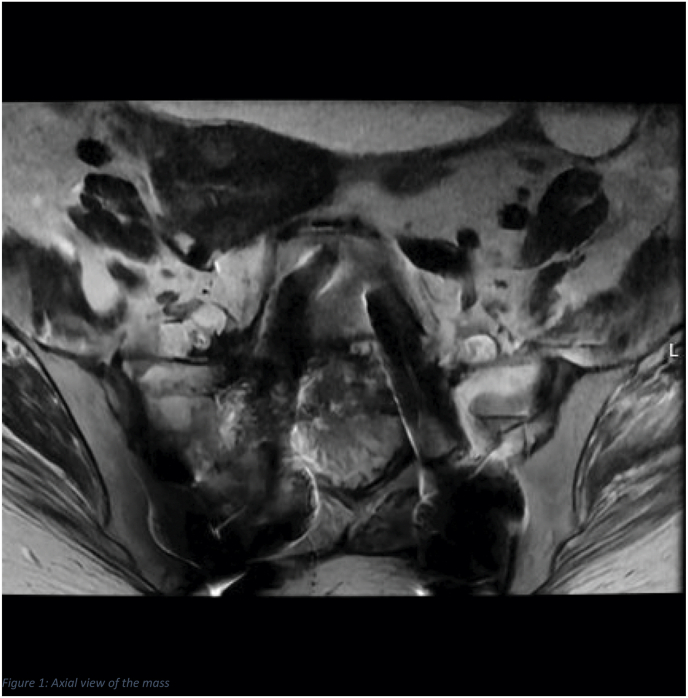
Axial view of the mass.

**Fig. 2:**
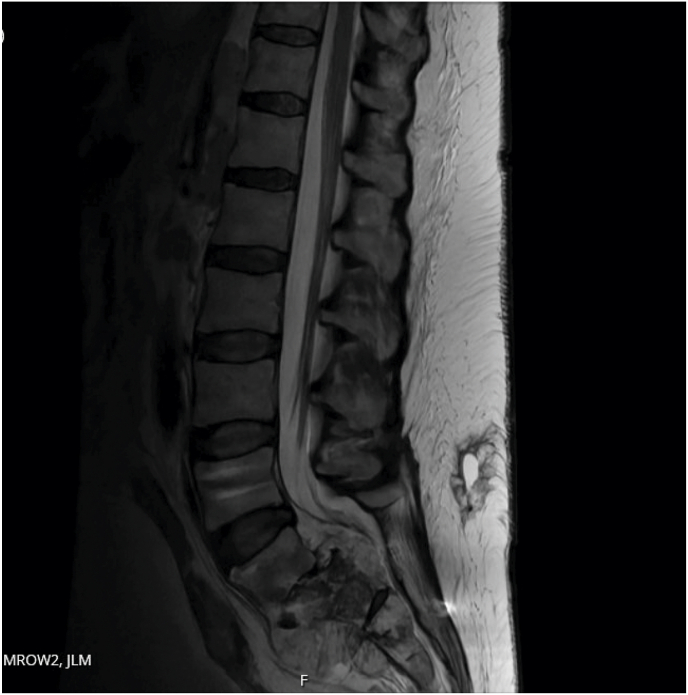
Sagittal view of the vertebral destruction with extrusion into the canal.

We present a 55-year-old female with metastatic colon cancer who was hospitalized for severe lower lumbar and bilateral radicular pain. This patient was unable to ambulate more than two to three steps, limited by extreme pain and subjective weakness in her lower extremities. Imaging revealed a soft tissue mass at S1, extending into the vertebral body and posterior epidural space with neuroforaminal involvement at L5-S1(Fig. 1, Fig. 2). During her inpatient stay, she was being managed with an escalating regimen of opioids with little to no improvement in her symptoms but with increasing adverse effects of somnolence—she was essentially sedated.

**Fig. 3:**
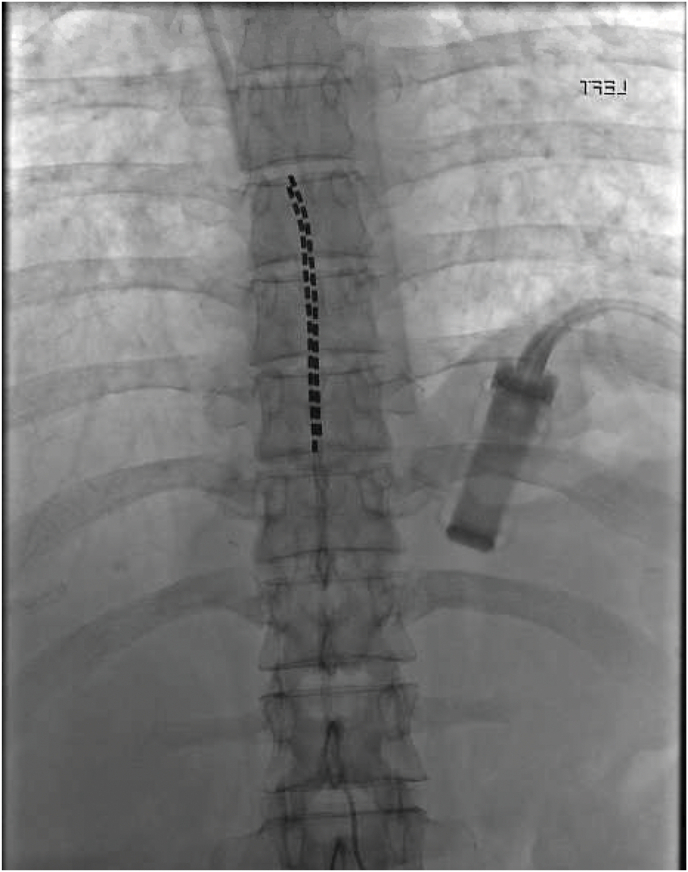
Fluoroscopic images of spinal cord stimulator trial leads spanning the vertebral body of T7.

The Palliative and Pain service were consulted for assistance with inpatient management of her intractable pain crisis. The patient was discussed at the weekly multidisciplinary inpatient palliative pain conference, and it was agreed upon to proceed with a collaborative inpatient spinal cord stimulator trial between the interventional pain and interventional radiology teams. Intrathecal pump was discussed but the patient did not have reliable transportation and lived far away from the academic medical center, making refills and troubleshooting difficult to manage in the long-term. The patient was scheduled for trial and under fluoroscopic guidance, 16-contact spinal cord stimulator leads were placed spanning the T7 vertebral bodies (Fig. 3). During programming, our patient preferred tonic stimulation over high frequency and other waveforms. The procedure was tolerated well without complications and patient was discharged to home.

**Fig. 4:**
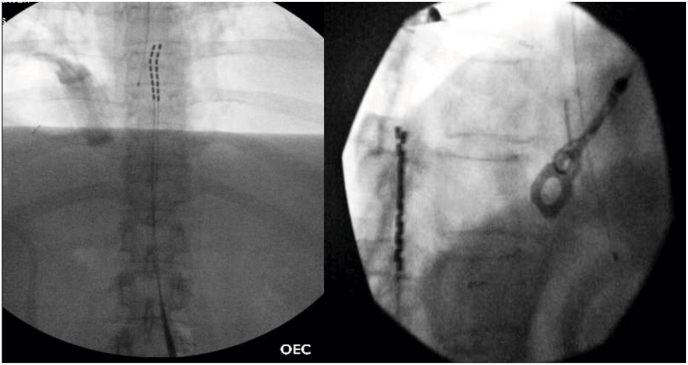
AP and lateral fluoroscopic imaging of spinal cord stimulator permanent leads spanning T7 vertebral body.

During the immediate post-op period, the patient had immediate pain relief and her opioid requirement was reduced from *500 morphine milliequivalents (MME) to* 200 MME. The patient was discharged to her home and over the next few days, she tolerated transitioning from intravenous opioid therapy to an oral regimen. She improved from a bedridden state to ambulating distances over 500 feet *without pain*, thus she was scheduled for permanent implant 10 days post-discharge (Fig. 4). The leads were placed under fluoroscopic guidance, spanning across the T7 vertebral body. She has not had any admissions for pain control since her implant and her pain continues to be stable and managed with a 2mg oral hydromorphone as needed.

## 3 Discussion

Colorectal cancer (CRC) is one of the most common cancers worldwide and the third ranked by mortality. 20 % of patients have metastatic disease at the time of diagnosis while another 25 % who present with localized disease will advance to metastases [11]. The liver and lungs are common site for metastatic disease and up to 27 % will develop bone metastases with the most common location being the vertebral column. With innovations in neoadjuvant treatment regimens and development of targeted therapies, the overall prognosis of CRC has improved leading to an increase in 5-year survival rates. However, with the increased median survival comes the consequences of not only a higher risk of developing bone metastases but also a higher risk of experiencing complications from the metastases such as metastatic bone destruction causing pain, pathological fractures, spinal cord compression or hypercalcemia.

Malignancy-related pain remains as one of the most frequently reported symptoms amongst patients with advanced stage cancer, in fact, almost 70 % of cancer patients suffer from pain and, for approximately one-third of this population, their pain is insufficiently controlled [12]. This unfortunate circumstance is due to many reasons notably poor communication, ineffective treatments and limited understanding of multimodal management. In the 1990s, opioids became the default choice for the treatment of all types of pain which inevitably led to the opioid epidemic that has caused everlasting damage to individuals, their families and society. Consequently, the importance of prioritizing research on non-opioid alternatives became clear and recent advancements in neuromodulation have offered viable options.

Tonic SCS mainly acts via a segmental spinal mechanism where it induces GABA-release from inhibitory interneurons in the spinal dorsal horn. Tonic SCS concurrently initiates neuropathic pain modulation through a supraspinal-spinal feedback loop and serotonergic descending fibers [13]. Spinal cord stimulation (SCS) has emerged as an effective treatment for chronic pain disorders; however, its precise mechanism of action remains to be fully elucidated. A prevailing hypothesis posits that SCS modulates neuronal activity in the spinal cord, particularly the dorsal horn, by activating large-diameter Aβ fibers, consequently inhibiting pain transmission from smaller diameter Aδ and C fibers via the gate control [7,10]. Moreover, SCS has been shown to influence the release of neurotransmitters, such as gamma-aminobutyric acid (GABA), serotonin, and norepinephrine, which modulate pain transmission [14,15]. Additionally, SCS has been implicated in activating endogenous pain inhibition systems, including the release of endogenous opioids and the activation of descending noradrenergic and serotonergic pathways [16]. Further research is warranted to fully comprehend the neurophysiological and molecular mechanisms underlying SCS to optimize patient outcomes and broaden its clinical applications.

One of the main benefits of considering SCS as a treatment option for patients with chronic pain syndromes is the possibility to decrease a patient's reliance on opiods and achieve long term pain relief. Gee et al. performed a prospective cohort study in patients undergoing SCS placement for various chronic pain conditions. They found 64 % of patients who required opioids for treatment of their pain syndromes prior to SCS implantation either reduced or eliminated opioid use completely at the one-year post SCS implantation follow-up [17]. In addition to decreasing the reliance on opioids, SCS also offers a potential solution for complete, long-term resolution of pain in patients with refractory, chronic pain. One study found 22 % complete remission, defined as greater than 80 % improvement in the Numeric Pain Rating Scale, in patients at the one-year mark post SCS implantation [18]. This remission was correlated with substantial increases in functional measures and overall quality of life improvements. Preoperative comorbidities and opioid use were associated with lower rates of complete remission. It is becoming increasingly important for physicians to responsibly manage the reliance on opioids to treat patients and SCS offers a novel way to not only achieve decreased opioid requirements but also possible remission.

As advancements are made in treatment modalities for colorectal cancer, the prolonged survival rate comes with the need for further progression in cancer-related pain management. This common complication of advanced disease remains a clinical challenge and requires multidisciplinary care and longitudinal follow-up. This case highlights SCS as a potential alternative to high dose opioids during a refractory pain crisis secondary to a radiculopathy in the setting of metastases. SCS may be useful for patients who are not appropriate candidates for opioid pain medications or more invasive interventional procedures due to comorbidities or an immunocompromised state. Unmanaged pain, contributes to approximately 21 % of hospital readmissions due to disease progression while our patient progressed to home with oral analgesics as needed [19]. Furthermore, the decrease in the patient's opioid requirements and increase in functional mobility provided significant improvement in quality of life, warranting further research regarding this novel indication in regards to its cost-effectiveness compared to traditional management. While we present a compelling case report, our limited sample prompts the need for further research with randomized trials and larger clinical samples are needed to appropriately test the effectiveness of SCS as a viable treatment option for acute cases of radiculopathy such as the one presented here.

## Conflict of interest

None.

## Disclosure of funding

None.

## Declaration of competing interest

The authors declare that they have no known competing financial interests or personal relationships that could have appeared to influence the work reported in this paper.
